# Knee-related disability was largely influenced by cognitive factors and disturbed body perception in knee osteoarthritis

**DOI:** 10.1038/s41598-021-85307-1

**Published:** 2021-03-12

**Authors:** Tomohiko Nishigami, So Tanaka, Akira Mibu, Ryota Imai, Benedict Martin Wand

**Affiliations:** 1 Department of Physical Therapy, Faculty of Health and Welfare, Prefectural University of Hiroshima, 1-1 Gakuen-tyou, Mihara, Hiroshima 723-0053 Japan; 2 Department of Rehabilitation, Fukuoka Orthopaedic Hospital, 2-10-50 Yanagikawachi, Minami-ku, Fukuoka, Fukuoka 815-0063 Japan; 3 Department of Physical Therapy, Konan Women’s University, 6-2-23 Morikita-machi, Higashinada-ku, Kobe, Hyogo 658-0001 Japan; 4 School of Rehabilitation, Osaka Kawasaki Rehabilitation University, 158 Mizuma, Kaizuka, Osaka 597-0104 Japan; 5 School of Physiotherapy, The University of Notre Dame Australia, 32 Mouat St, Fremantle, WA 6160 Australia

**Keywords:** Musculoskeletal system, Disability, Pain, Osteoarthritis, Rehabilitation

## Abstract

The aim of this study was to explore the existence of subgroups in a cohort of people with knee osteoarthritis (OA) based upon data from multiple pain-related variables and to profile identified clusters according to levels of pain intensity and knee-related disability. Three hundred and three people with knee OA were recruited. Latent profile analysis was used to confirm the optimal number of knee OA subgroups. Body mass index, radiographic knee OA severity, pain catastrophizing, pain related self-efficacy, and knee specific self-perception, were incorporated into the model. Cluster, demographic and clinical variables were compared between the resulting classes. Four distinct classes were identified. Cluster 1 (28.7%) represented early radiographic OA, and moderate pain intensity, disability and cognitive and perceptual dysfunction. Cluster 2 (18.8%) showed advanced radiographic OA, and moderate pain intensity, disability and cognitive and perceptual dysfunction. Cluster 3 (34.3%) represented various levels of radiographic OA, and the lowest pain intensity, disability and cognitive and perceptual dysfunction. Cluster 4 (18.1%) represented various levels of radiographic OA, the highest disability and cognitive and perceptual dysfunction. Considering cognitive factors and disturbed body perception may help to explain the phenomenon of the discrepancy between the knee-related disability and the severity of radiographic knee OA.

## Introduction

Knee osteoarthritis (OA) is a common diagnosis in people with knee pain and is related to various clinical symptoms, such as pain, swelling, feelings of stiffness, problems moving the joint, confidence in loading the knee and difficulties performing activities of daily living, all of which can influence health-related quality of life^1–3^. However, it is well known that there is a discrepancy between clinical symptoms and radiographic evidence of joint degeneration in people diagnosed with knee OA^4–6^, particularly when differentiating between the painful and non-painful knee in the same individual^7^.

It is likely that factors other than osteoarthritic changes within the knee contribute to the clinical presentation in those with knee OA. For example, people with higher Body Mass Index (BMI) experience greater pain than individuals with lower BMI even when taking into account OA severity^8^. Also, a recent systematic review demonstrated that there was moderate evidence for a relationship between pain intensity and cognitive factors such as self-efficacy, somatization and pain catastrophising in people with knee OA^9^. Furthermore, preliminary data indicates that findings thought to be associated with disturbed body-perception such as reduced tactile acuity^10^, impairments in motor imagery performance^11^, and degraded proprioceptive acuity^12,13^ were also associated with clinical status in people with knee OA. Recently, the Fremantle Knee Awareness Questionnaire (FreKAQ) was developed to directly assess body-perception specific to the knee in people with knee OA^14^ by modifying Fremantle Back Awareness Questionnaire (FreBAQ)^15^ and it appears that self-reported disruption of knee perception is also associated with pain intensity and disability in this population^14^. These findings suggest that the clinical status of people with knee OA is contributed to by features across the biopsychosocial spectrum. It is likely that a fuller understanding of the pain experience in those with knee OA will come from greater understanding of how these various factors coalesce in their contribution to clinical status.

There have been several attempts to look at how multiple factors interact in people with knee OA by using various types of sub-group analyses. Finan et al.^16^ constructed four subgroups by dichotomizing pain intensity (median split) and knee OA severity (grades I-II versus III-IV) and showed that quantitative sensory testing and psychological distress differed across the four resultant subgroups. Ozcakir et al.^17^ grouped patients into early radiographic OA and advanced radiographic OA groups and investigated differences in the clinical profile of these two groups across biological, psychological and social domains. Other studies have attempted to divide the knee OA population into subgroups utilizing variables such as knee OA severity, BMI or psychological status using traditional cluster analysis^18^ and latent cluster analysis^19^ and have found evidence for distinct clinical phenotypes amongst those with knee OA. For example, Cruz-Almeida et al.^20^ demonstrated that groupings based on psychological characteristics produced four distinct clusters that displayed unique sets of clinical and somatosensory features. However, issues around the interaction between clinical status and severity of structural changes are still not fully resolved and no previous study has considered the role of body perception in these investigations. We were particularly interested in the influence of pain related cognitions and how the painful knee was perceived by the individual on functional status. Firstly, because these are factors amenable to treatment, and secondly, it is plausible that how the knee feels to the person, the confidence they have in using the knee and their thoughts about the meaning and controllability of pain are potentially self-reinforcing and interact to determine the level of functional capacity independent of disease severity.

To address these issues we used latent profile analysis (LPA) of multiple biological, psychological and perceptual variables to explore the existence and number of subgroups in a cohort of people with knee OA and to profile identified clusters according to pain intensity and levels of knee-related disability. We hypothesize a likely three class model with low, moderate, and high levels of pain and disability that would be mirrored by the degree of the cognitive and perceptual dysfunction, regardless of the severity of structural changes.

## Results

All characteristics are summarized in Table 1. The pain intensity was significantly correlated with, OKS (ρ = − 0.38), BMI (ρ = 0.25), Kellgren/Lawrence scale (K/L scale) (ρ = 0.19), Pain Catastrophizing Scale (PCS) (ρ = 0.29), Pain Self-Efficacy Questionnaire (PSEQ) (ρ = − 0.24), and FreKAQ (ρ = 0.29) (P < 0.01 for all). Oxford Knee Score (OKS) was significantly correlated with, BMI (ρ = − 0.21), K/L scale (ρ = − 0.33), PCS (ρ = − 0.60), PSEQ (ρ = 0.50), and FreKAQ (ρ = − 0.59) (P < 0.01 for all).

**Table 1:** Characteristics of this study population.

Variable		All (n = 303)
Demographic variables	Age (year)	69.1 (9.9)
	Sex male/female (n)	66/237
	Pain duration (week)	19.2 (56.3)
Cluster variables	BMI (kg/m^2^)	23.6 (3.0)
	KL I (n)	69
	II (n)	128
	III (n)	73
	IV (n)	33
	PCS (0–52)	23.0 (11.2)
	PSEQ (0–60)	39.8 (12.7)
	FreKAQ (0–36)	12.5 (7.8)
Clinical variables	Pain intensity (0–10)	4.5 (2.1)
	OKS (0–48)	33.30 (8.8)

Values are the mean ± SD.

*BMI* body mass index, *KL* Kellgren/Lawrence, *OKS* Oxford knee score, *PCS* pain catastrophizing scale, *PSEQ* Pain self-efficacy questionnaire, *FreKAQ* Fremantle knee awareness questionnaire.

### Determination of class structure

We performed the LPA for two to six clusters (Table 2). The 7-class model did not outperform the 6-class model according to LMR-LRT (p = 0.50). AIC and SSABIC improved gradually as the number of clusters increased. The 5-class model seems to be a better fit than a 4-class model according to AIC, SSABIC, and LMR-LRT. However, the smallest class in the 5-class model represented only 0.6% of the study population (2 participants of the 303 available). In contrast, the smallest class in the 4-class model represented 18.2% of the study population. Therefore, we concluded that a 4-class model provided the best fit to the data.

**Table 2:** Fit indicators for the 2 to 6 latent profile solutions.

Model	AIC	ABIC	Entropy	LMR-LRT	Value
2	9002.308	9010.984	0.713	< 0.0001	211.430
3	8949.695	8961.625	0.767	0.0015	62.781
4	8927.998	8943.181	0.739	0.0469	32.743
5	8910.211	8928.648	0.785	0.0024	28.943
6	8891.701	8913.396	0.789	0.50	29.641

*AIC* Aikake Information Criterion, *ABIC* Sample-size adjusted Bayesian Information Criterion, *LMR-LRT* Lo-Mendell-Rubin Likelihood Ratio Test.

### Comparison of classes

Differences in clusters variables are detailed in Table 3. There was no significant difference in BMI across all clusters. There was no statistically significant difference in radiographic knee OA severity between the cluster 1 and 3 (p = 0.04), but significant differences between the other clusters (p < 0.001 for all). Clusters 1 and 3 represented mainly early structural changes, whereas cluster 2 contained people with mainly late changes and cluster 4 a mix of both. There was no significant difference in PCS scores between clusters 1 and 2 (p = 0.12). There were significant differences between the other clusters (p < 0.001 for all) with cluster three recording very low levels of catastophisation and cluster 4 high levels. PSEQ scores demonstrated a similar pattern, clusters 1 and 2 were not different (p = 0.26) though there were differences between the other groups (p < 0.001 for all). Cluster 3 participants reported feeling confident to perform activities when in pain whereas people in cluster 4 were not confident. FreKAQ scores were also no different between cluster 1 and 2 (p = 1.00) with people in both groups reporting a moderate level of perceptual disturbance. There were significant differences between the other clusters (p < 0.001 for all) with again cluster three showing low levels of perceptual dysfunction and cluster 4 representing substantial perceptual dysfunction. The psychological and perceptual measures behaved very similarly across the three clusters whereas the degree of degeneration shows a quite different pattern and BMI was near identical in all subgroups.

**Table 3:** Descriptive statistics of the 4 clusters derived from cluster variable.

Variable	Class 1 n = 87 (28.7%)	Class 2 n = 57 (18.8%)	Class 3 n = 104 (34.3%)	Class 4 n = 55 (18.1%)	Effect size
BMI (kg/m^2^)	23.1 (2.9)	24.2 (2.8)	23.4 (2.9)	24.2 (3.5)	η^2^ = 0.02
KL I (n)	34**^,†^	0*^,^***^,†^	34**^,†^	1*^,^**^,^***	V = 0.48
II (n)	53	0	55	20	
III (n)	0	32	14	27	
IV (n)	0	25	1	7	
PCS (0–52)	26.9 (7.1)***^,†^	24.0 (7.4)***^,†^	12.5(7.1)*^,^**^,†^	36.0 (7.7)*^,^**^,^***	η^2^ = 0.58
PSEQ (0–60)	35.6 (9.6)***^,†^	39.1 (10.1)***^,†^	49.8 (9.0)*^,^**^,†^	28.4 (12.1)*^,^**^,^***	η^2^ = 0.39
FreKAQ (0–36)	13.0 (4.5)***^,†^	13.3 (4.4)***^,†^	5.4 (4.0)*^,^**^,†^	24.4 (4.0)*^,^**^,^***	η^2^ = 0.71

Values are the mean ± SD.

*BMI* body mass index, *KL* Kellgren/Lawrence, *PCS* Pain Catastrophizing Scale, *PSEQ* Pain Self-Efficacy Questionnaire, *FreKAQ* Fremantle Knee Awareness Questionnaire.

*Indicates significant difference compared with Class 1.

**Significant difference compared with Class 2.

***Significant difference compared with Class 3.

^†^Significant difference compared with Class 4.

P values of less than 0.012 for Bonferroni post hoc tests and Chi-squared test, and 0.0083 for Wilcoxon rank sum.

Differences in demographic variables and external measures are detailed in Table 4. Cluster 3 had significantly more males than cluster 4 (p < 0.001).

**Table 4:** Descriptive statistics of demographic variables and external variables.

Variable	Class 1	Class 2	Class 3	Class 4	Effect size
Age (year)	66.4 (8.9)**	73.8 (8.5) *^,^***	67.7 (10.4)**	70.9 (9.8)	η^2^ = 0.07
Sex male/female (n)	20/67	12/45	32/72^†^	2/53***	V = 0.23
Pain intensity (0–10)	4.5 (2.0)***	5.1 (2.0)***	3.6 (2.1) *^,^**^,†^	5.4 (1.6) ***	η^2^ = 0.10
OKS (0–48)	32.9 (6.5)***^,†^	29.7 (7.9)***^,†^	39.7 (6.3) *^,^**^,†^	24.2 (7.3) *^,^**^,^***	η^2^ = 0.40

*OKS* Oxford Knee Score.

*Indicates significant difference compared with Class 1.

**Significant difference compared with Class 2.

***Significant difference compared with Class 3.

^†^Significant difference compared with Class 4.

P values of less than 0.012 for Bonferroni post hoc tests and Chi-squared test, and 0.0083 for Wilcoxon rank sum.

Pain intensity scores were significantly lower in group 3 in comparison to all other groups (p < 0.001 for all). There was no significant difference in OKS between cluster 1 and 2 (p = 0.046), but the differences between other clusters was significant (p < 0.001). Cluster 3 had significantly better level of function than the other three groups with similar favorable scores for cognitions and body perception and the lowest level of pain intensity yet they did not have the most favorable structural profile and BMI was the same. Cluster 4 had significantly worse function than the other three groups (all p < 0.001), the cognitive and perceptual scores were also the most maladaptive yet pain intensity was similar to groups 1 and 2 and they did not have the worst degeneration profile.

## Discussion

This study explored clinical subgroups in knee OA differentiated by BMI, knee OA severity, pain related cognitions and disturbed body perception. We hypothesized a likely three class model with low, moderate, and high level of pain and disability that would be mirrored by the degree of the cognitive and perceptual dysfunction, regardless of the severity of structural changes. The results support our hypothesis to some degree. Four distinct classes were identified in this study as follows. Cluster 1: a group of people with mild radiographic OA, and moderate levels of cognitive and perceptual dysfunction. This group demonstrated moderate levels of pain and disability. Cluster 2: a group of people with advanced radiographic OA and moderate levels of cognitive and perceptual dysfunction. Moderate levels of pain and disability were also seen in this group. Cluster 3: a group of people with various radiographic findings and minimal perceptual and cognitive dysfunction. This group had the lowest levels of pain and disability. Cluster 4: a group of people with various radiographic findings, and the most adverse perceptual and cognitive profile. This group reported the highest level of disability. BMI was the same across all four subgroups.

Several studies have used cluster analysis to identify subgroups within the knee OA population. The cluster analysis carried out by Kittelson et al.^19^ yielded four clusters including a group with a greater number of health co-morbidities, a group with high levels of knee joint tenderness and quadriceps weakness, another with high levels of psychological distress and widespread pain and a final group with mild OA, low levels of distress, few comorbidities and good quadriceps function^19^. Pain and disability demonstrated the same pattern across the various groups, and those in the psychological distress cluster demonstrated the highest levels of pain and disability^19^. Knoop et al.^18^ reported five clusters in their analysis of 842 OA knee patients including a minimal joint disease cluster, a strong muscle cluster, a non-obese and weak muscle cluster, an obese and weak muscle cluster and a depressive cluster. Again pain and disability varied in a similar way across groups and further support was provided for the importance of psychological factors with the depressed group demonstrating the worst clinical profile. Our study suggests a similar influence of psychological function on clinical status, though with some differential effects on pain and disability noted. This is the first study that has integrated assessment of disturbed body perception into a cluster analysis. The group with the greatest level of disturbed self-perception and cognitive dysfunction were the most disabled though they did not have the worst degeneration profile and demonstrated similar pain intensity scores to all but one of the groups. This finding hints that in some people confidence in the knee, pain cognitions and perceptual awareness of the knee interact to shape functional capacity more than disease severity or pain intensity.

The findings reported here offer some insight into the often reported discrepancy between the level of structural changes and functional capacity. Cluster 1 and 3 show similar knee OA severity, yet cluster 1 has significantly higher disability. This might be explained by the greater cognitive and perceptual dysfunction seen in cluster 1, particularly as pain intensity is not significantly different between these two groups. Clusters 1 and 2 differ starkly in their level of structural change yet have similar pain intensity and disability levels. This discrepancy between structural pathology and clinical status might be explained by the similarities these two groups demonstrate in cognitive and perceptual function. In both these comparisons disability maps to cognitive and perceptual function, not radiographic status. Radiographic changes on imaging might be the defining feature of knee OA but these data suggest the functional impact on the individual is more related to their thoughts about the meaning of pain, the confidence they have in their knee and how the knee feels to them.

This has important implications for the care pathways offered to people with knee OA and the content of non-surgical care. Total knee arthroplasty is often seen as the definitive solution for those with late stage degeneration^21^, despite there being a significant proportion of people reporting on-going pain post arthroplasty^22^. One possible interpretation of these data is that surgery might best be indicated for those with late degeneration and low levels of cognitive and perceptual impairment. Structural pathology, the putative target of surgery, might be the primary contributor to clinical status in this clinical phenotype. Alternatively, for those with late degeneration and cognitive and perceptual dysfunction, education and rehabilitation to address these issues might be a better option, or their attendance prior to surgery might improve on the success rate of knee arthroplasty, particularly as previous research suggests that preoperative pain catastrophizing^23^ and pain self-efficacy^24^ are important in understanding outcomes after total knee arthroplasty. Clearly more data is needed to explore these ideas.

There is some support for rehabilitation in the management of knee OA^25^, though effect sizes are limited^26^. Rehabilitation generally involves exercises to improve the capacity of lower limb muscles, strategies to increase general physical activity, advice on lifestyle modification and education regarding the condition^27,28^. This study provides further support for the idea that the educational component should address maladaptive pain related cognition^29^, and there are groups for whom this is particularly important. The effect sizes seen with rehabilitation might also be improved with the inclusion of approaches that target body perception such as sensorimotor training^30^. This would likely most apply those people represented by Cluster 4, the group with the lowest functional capacity, and a recent hypothesis generating study by our group provides support for the idea of an interaction between conservative treatment success and disrupted body perception^31^. While comparison between individual scales is not straightforward, this group appears to have particular problems with knee specific body perception. The difference between the average scores in cluster 4 and the mean score in the lowest group differ by around 2 and 3 standard deviations for the PSEQ and PCS respectively, yet the average FreKAQ score for those in cluster 4 is 4.5 standard deviations higher than the value recorded in the cluster with the lowest score. Further research of the response of this patient phenotype to treatment targeted at body perception would be particularly interesting.

The present findings should be considered in light of the limitations of the study. Firstly, as with all studies of this sort, analysis is limited by the variables assessed. We limited our investigation to a handful of biopsychosocial variables that are easily measured in the clinic and offer some targets for rehabilitation. Clinical measures of muscle strength and depressive symptoms may have offered greater insight into clinically useful phenotypes amongst people with knee OA. We did not find a difference in BMI between groups. This might reflect some unique features in our sample. The average BMI (23.6 ± 3.0 kg/m^2^) and obesity rate (4.2%) in this study was lower than those of a previous study (29.9 ± 4.8 kg/m^2^, 46%, respectively) in which obesity status contributed to the cluster analysis^18^. Although obesity is considered as one of the treatment targets to reduce knee OA symptoms^32,33^, this might be of less importance in the Japanese population. Some differences in age and gender are apparent across the groups and may impact on some of the differences observed, though differences in neither of these non-modifiable factors seems to closely track other considered variables. Lastly, this study did not include follow-up, therefore, it is not possible to comment on the stability of the class structure or offer any insight into variations in clinical trajectories that might be apparent within the clusters identified.

## Conclusions

Our results demonstrated that knee-related disability was strongly influenced by pain related cognitive factors and disturbed body perception. Considering pain cognitions and disturbed body perception may help to explain the phenomenon of the discrepancy between the knee-related disability and radiographic knee OA and offer some suggestions for management of people with knee OA.

## Methods

Ethical approval was obtained from the institutional ethics committee of Kyushu Medical Sports Vocational School. Written, informed consent was obtained from all participants prior to the study. The study was conducted in compliance with the Declaration of Helsinki.

### Participants

Three hundred and three people with symptomatic knee OA were recruited from thirteen orthopedic hospitals. All patients underwent an X-ray examination and were screened and recruited by orthopedists, who confirmed the presence of knee joint pain. Inclusion criteria were adults with unilateral, symptomatic and radiographic knee OA (a score of at least one on the K/L scale). The exclusion criteria were total knee arthroplasty, serious pathologies (unhealed fractures, tumors, acute trauma, or serious illness), neurological findings (muscle weakness, loss of sensation or reflexes), and diagnosed psychiatric disorders.

### Procedure

Demographic data (age, gender), BMI, severity of structural changes, pain-related catastrophizing, pain-related self-efficacy, knee-specific body-perception, pain duration, pain intensity and knee pain-related disability were assessed in all participants.

Severity of structural changes was evaluated using the K/L scale, which is a method of classifying the severity of knee OA using a five points scale (0–IV)^34^. Those classified as level one and two were grouped as ‘early’ and classifications three and four as ‘advanced’ radiological OA for analysis. Pain-related catastrophizing was measured using the Japanese version of the PCS^35^. The scale comprises 13 items related to magnification, rumination, and helplessness about pain with higher scores indicating greater levels of pain related catastrophisation^36^. The Japanese version of the PCS has been confirmed internal consistency and criterion validity^32^. The Japanese version of the PSEQ was used to assess the confidence people with knee pain have in performing activities while in pain^37^. The Japanese version of the PSEQ demonstrated excellent internal consistency, criterion validity, and test–retest reliability^37^. Higher scores of the PSEQ indicate higher levels of confidence^38^. Self-reported body-perception of the knee was evaluated using the FreKAQ^14^. The FreKAQ is composed of nine items that relate to neglect-like symptoms (e.g. I feel like my knee is not part of my own body), reduced proprioceptive acuity (e.g. When performing activities of daily living (housework, work, etc.), I do not know how much my knee is moving), and perceived body part shape and size (I feel like my knee is bigger (swollen)). A 5-point response scale (range: 0 = “never” up to 4 = “always”) was used to enable quantitative assessment of any reported symptoms. Higher scores on the FreKAQ indicate more disturbed body perception. Pain intensity during movement was based on recall and measured using a 0–10 numeric rating scale anchored at the left with “0 = no pain” and at the right with “10 = unbearable pain”. We chose pain intensity during movement as movement-evoked pain appears to be more relevant to understanding pain related disability than usual pain intensity^39,40^. Disability was measured using the Japanese-validated version of the OKS for people with knee OA^41,42^. The scale is scored from 0 to 48, with higher scores indicating better function.

### Sample size

Expert opinion on the use of Latent Profile Analysis (LPA) suggest that 300 participants are needed to perform an LPA^43^, therefore, our sample size was adequate for the analyses undertaken.

### Statistical analyses

SPSS 25.0 (IBM, Tokyo, Japan) and Mplus software (version 8.0, Muthen & Muthen, Los Angeles, CA, USA) were used to conduct the data analyses.

#### Preliminary analyses

Pearson’s correlations between pain intensity, disability and variables selected for LPA were computed to confirm whether our data were suitable to perform LPA.

#### Latent profile analyses

We utilized LPA to identify the optimal number of knee OA subgroups. This technique has been found to behave efficiently for the discrimination phase and allow the computation of posterior membership probabilities, which, in turn, allow the statistical comparison of the resulting profiles^44,45^. BMI, K/L grade, PCS, PSEQ, and FreKAQ were incorporated into the model. The Akaike information criteria (AIC), sample size adjusted Bayes Information Criteria (SSABIC), Entropy, and Lo-Mendell-Rubin Adjusted Likelihood Ratio Test (LMR-LRT)^46^ were used to determine the number of classes that best fit the data. Smaller values of AIC and BIC indicate better fit. Entropy values close to 1 indicate class distinctiveness^47^. LRT indicates the significance of improvement in model fit of the number of groups being tested versus one less group. Optimal number of knee OA subgroups was determined from the combination of the lowest BIC with the highest LRT and Entropy.

To detect potential cluster group differences in demographic variables (age and sex), cluster variables (BMI, K/L grade, PCS, PSEQ, and FreKAQ), and external measures (pain intensity and disability), 1-way ANOVAs with Bonferroni post hoc tests, Wilcoxon rank sum test and Chi-squared test were used. P values of less than 0.012 for Bonferroni post hoc tests and Chi-squared test, and 0.0083 for Wilcoxon rank sum test were considered statistically significant. Effect sizes were calculated based on η^2^ (a large effect was defined as > 0.14, a moderate effect as 0.06 to 0.14, and a small effect as < 0.06), V (a large effect was defined as > 0.5, a moderate effect as 0.3 to 0.5, and a small effect as < 0.3).

## Data Availability

The datasets generated and analysed during the current study are available from the corresponding author on reasonable request.
